# Identification and Functional Analysis of G Protein-Coupled Receptors in 20-Hydroxyecdysone Signaling From the *Helicoverpa armigera* Genome

**DOI:** 10.3389/fcell.2021.753787

**Published:** 2021-10-26

**Authors:** Yan-Li Li, Yan-Xue Li, Xiao-Pei Wang, Xin-Le Kang, Ke-Qin Guo, Du-Juan Dong, Jin-Xing Wang, Xiao-Fan Zhao

**Affiliations:** Shandong Provincial Key Laboratory of Animal Cells and Developmental Biology, School of Life Sciences, Shandong University, Qingdao, China

**Keywords:** genome, GPCR, 20-hydroxyecdysone (20E), forkhead box O, *Pten*

## Abstract

G protein-coupled receptors (GPCRs) are the largest family of membrane receptors in animals and humans, which transmit various signals from the extracellular environment into cells. Studies have reported that several GPCRs transmit the same signal; however, the mechanism is unclear. In the present study, we identified all 122 classical GPCRs from the genome of *Helicoverpa armigera*, a lepidopteran pest species. Twenty-four GPCRs were identified as upregulated at the metamorphic stage by comparing the transcriptomes of the midgut at the metamorphic and feeding stages. Nine of them were confirmed to be upregulated at the metamorphic stage. RNA interference in larvae revealed the prolactin-releasing peptide receptor (PRRPR), smoothened (SMO), adipokinetic hormone receptor (AKHR), and 5-hydroxytryptamine receptor (HTR) are involved in steroid hormone 20-hydroxyecdysone (20E)-promoted pupation. Frizzled 7 (FZD7) is involved in growth, while tachykinin-like peptides receptor 86C (TKR86C) had no effect on growth and pupation. Via these GPCRs, 20E regulated the expression of different genes, respectively, including *Pten* (encoding phosphatidylinositol-3,4,5-trisphosphate 3-phosphatase), *FoxO* (encoding forkhead box O), *BrZ7* (encoding broad isoform Z7), *Kr-h1* (encoding Krüppel homolog 1), *Wnt* (encoding Wingless/Integrated) and *cMyc*, with hormone receptor 3 (HHR3) as their common regulating target. PRRPR was identified as a new 20E cell membrane receptor using a binding assay. These data suggested that 20E, via different GPCRs, regulates different gene expression to integrate growth and development.

## Introduction

G protein-coupled receptors (GPCRs) are present widely in animals and humans (Hanlon and Andrew, 2015). GPCRs sense and transmit external stimuli into cells to regulate a variety of physiological processes, including cognition, metabolism, inflammation, immunity, and cell proliferation (Rasmussen et al., 2011). There are more than 800 GPCRs encoded in the human genome (Fredriksson and Schioth, 2005), over 1,300 GPCRs in mice, 116 classical GPCRs, which can act as guanine nucleotide exchange factors (GEFs), in *Drosophila* (Hanlon and Andrew, 2015), and 276 in *Anopheles gambiae* (Hill et al., 2002). The importance of GPCRs in cellular signaling has resulted in ∼34% of human drugs acting at GPCRs (Hauser et al., 2017). GPCRs are also suggested as targets for next generation pesticides (Audsley and Down, 2015). An interesting phenomenon in GPCRs-mediated signaling is that several GPCRs transmit the same signal of a ligand. For example, nine GPCRs function for adrenaline and five for dopamine (Hauser et al., 2017); however, the mechanism is unclear.

G protein-coupled receptors also transmit animal steroid hormone signals in the cell membrane. For example, GPCR 30 (GPR30/GPER) is an estrogen cell membrane receptor and transmits estrogen signals in mammals (Maggiolini and Picard, 2010). The dopamine/ecdysteroid receptor (DopEcR) transmits the non-genomic signal of insect molting hormone 20-hydroxyecdysone (20E) in *Drosophila* (Srivastava et al., 2005) and in *Helicoverpa armigera* (Kang et al., 2019). To date, several GPCRs have been proven to transmit 20E signals in *H. armigera* (Zhao, 2020), including ecdysone-responsible GPCR 1 (ErGPCR-1), ecdysone-responsible GPCR 2 (ErGPCR-2), and ecdysone-responsible GPCR 3 (ErGPCR-3) (Cai et al., 2014a; Wang et al., 2015; Kang et al., 2021). These data suggest that several GPCRs function as steroid hormone receptors; however, whether any other GPCRs transmit 20E signals, and the mechanism by which several GPCRs function in 20E signaling, are unclear.

*Helicoverpa armigera* is a widespread lepidopteran agricultural pest (Wu et al., 2008). We used *H. armigera* as the research model to identify the new GPCRs involved in 20E signaling and addressed the mechanism of their function in the 20E pathway. In all, 122 GPCRs were identified from the genome of *H. armigera*. Six GPCRs transmit 20E signal for hormone receptor 3 (*HHR3*) expression, a 20E-induced delayed early gene (Palli et al., 1997). 20E, via different GPCRs, regulates the expression of various genes, including *Pten* (encoding phosphatidylinositol-3,4,5-trisphosphate 3-phosphatase), *FoxO* (encoding transcription factor forkhead box O), which are known playing roles in 20E signaling (Cai et al., 2016), *BrZ7* (encoding broad isoform Z7), a transcription factor that promotes metamorphosis (Cai et al., 2014b), *Kr-h1* (encoding Krüppel homolog 1), the antimetamorphic effector induced by juvenile hormone (JH) (Belles, 2020), and *Wnt* (encoding Wingless/Integrated) and *cMyc*, which play significant roles in insect growth and development (Clevers, 2006; Kayukawa et al., 2012; Cai et al., 2014b), to integrate growth and metamorphosis. One GPCR, prolactin-releasing peptide receptor (PRRPR), was determined to bind 20E. Our study presents an example to explain the mechanism by which several GPCRs transmit the same signal.

## Results

### Identification of *Helicoverpa armigera* G Protein-Coupled Receptors From the Genome

**FIGURE 1:**
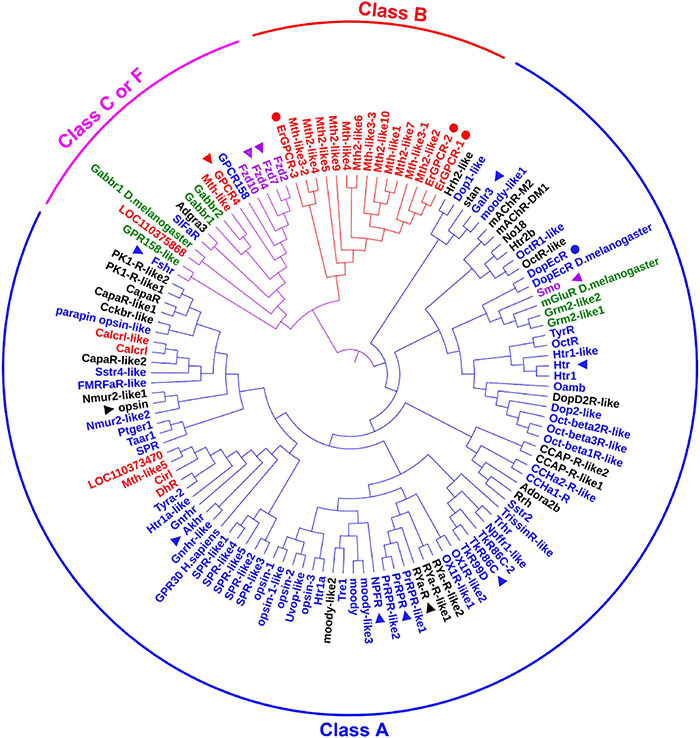
The phylogenetic analysis of classical GPCRs of *H. armigera*. Names in blue indicate GPCRs belonging to class A that were identified in the genome. Names in red indicate GPCRs belonging to class B that were identified in the genome. Names in green indicate GPCRs belonging to class C that were identified in the genome. Names in purple indicate GPCRs belonging to class F that were identified in the genome. Names in black indicate GPCRs that have not been classified in the genome. GenBank numbers were shown in Supplementary Table 1. The triangle represents the GPCRs studied in this article, and the circle represents the GPCRs in 20E signaling studied previously.

We searched for all GPCRs from the genome of *H. armigera* to identify classification of those GPCRs that are involved in 20E signaling. We found 122 genes encoding classical GPCRs in the *H. armigera* genome^1^ using BLAST (Basic Local Alignment Search Tool) with *Drosophila* and *Bombyx mori* GPCRs, respectively. Having removed four GPCRs with large sequence differences, the sequences of 118 presumed GPCRs, named as they are in the genome, were used to create a phylogenetic tree. These GPCRs could be divided into three clades according to four major categories of GPCRs (Sadowski and Parish, 2003): Class A (89 sequences), class B (15 sequences) and class C or F (14 sequences) (Figure 1 and Supplementary Table 1). Some GPCRs from *D. melanogaster* and *Homo sapiens* were used as landmarks of the classes, respectively. Fifteen GPCRs annotated to class A, B, C, or F in the genome were reclassified in different classes according to the sequences, which are marked with the related colors in each class in Figure 1. Twenty-five GPCRs that were not classified in the genome were gathered to different classes according to our phylogenetic analysis, which are marked in black in Figure 1. Four GPCRs known to transmit 20E signals in *H. armigera*, were classified as Class A (DopEcR) and class B (ErGPCR-1, ErGPCR-2, and ErGPCR-3).

### Screening of the G Protein-Coupled Receptors in 20E Signaling

**FIGURE 2:**
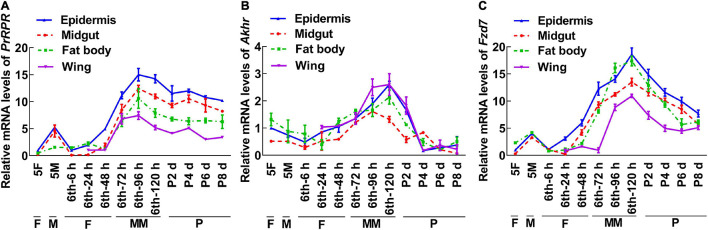
qRT-PCR showing the upregulated GPCRs during metamorphosis in all four tissues. **(A–C)** The relative mRNA levels of *PrRPR*, *Akhr*, and *Fzd7*. *Actb* was used as a control. All the experiments were performed in triplicate, and the bars indicate the mean ± SD. 5F, fifth instar feeding larvae; 5M, fifth instar molting larvae; 6th–6 h to 6th–120 h, time stages of sixth instar larvae; P2 d–P8 d, 2 day to 8-day-old pupae; F, feeding; M, larval molting; MM, metamorphic molting; P, pupae stage.

**FIGURE 3:**
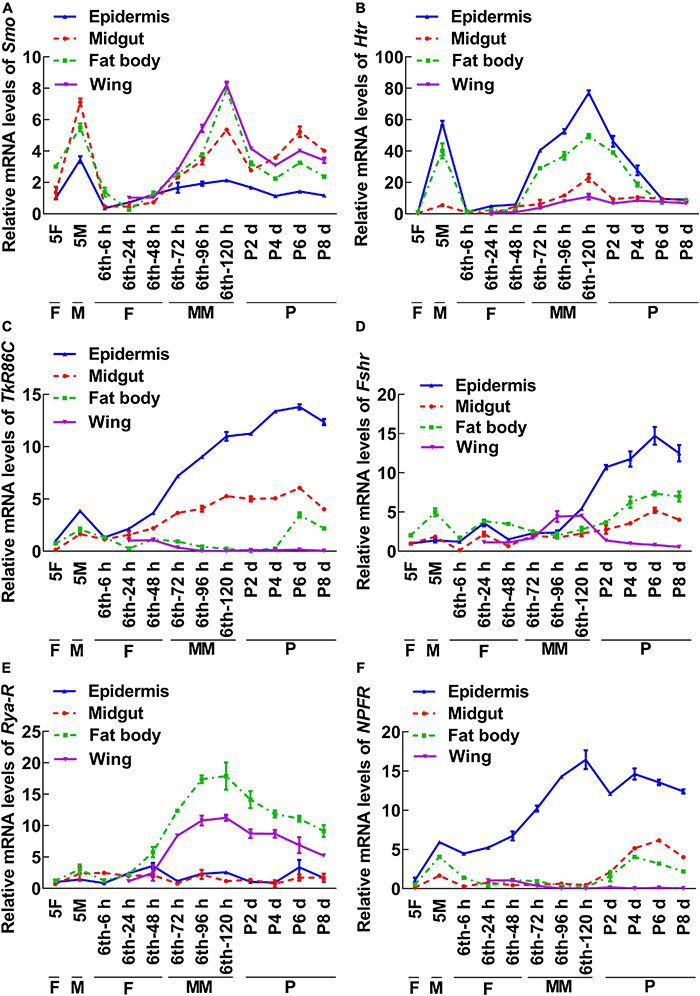
qRT-PCR showing the upregulated GPCRs during metamorphosis with tissue differences. The mRNA levels of GPCRs in *H. armigera* larval tissues. **(A–F)** The relative mRNA levels of *Smo, Htr, TrR86C, Fshr, Rya-R*, and *NPFR*. *Actb* was used as the control. All the experiments were performed in triplicate and the bars indicate the mean ± SD.

To screen for GPCRs involved in 20E signaling pathway, we compared the transcriptomes of the midgut at the feeding stage (6th–24 h) and the metamorphic molting stage (6th–72 h). Twenty-four GPCRs were found to be upregulated and seven were downregulated in the metamorphic stage (Supplementary Figure 1), suggesting that these twenty-four GPCRs are involved in metamorphosis. To examine the transcriptome analysis, 13 of the GPCRs (11 upregulated and 2 downregulated) from different classes were selected and examined for their developmental expression profiles in tissues using quantitative real-time reverse transcription PCR (qRT-PCR) to validate the result of the transcriptome analysis. Three GPCR genes, *PrRPR*, *Akhr* (encoding adipokinetic hormone receptor), and *Fzd7* (encoding frizzled 7), showed increased expression during metamorphosis (MM to P) in four detected tissues (Figure 2). Six GPCR genes, *Smo* (encoding smoothened), *Htr* (encoding 5-hydroxytryptamine receptor), *TkR86C* (encoding tachykinin-like peptides receptor 86C), *Fshr* (encoding follicle-stimulating hormone receptor), *Rya-R* (encoding the RYamide receptor), and *Npfr* (encoding neuropeptide F receptor) showed increased expression during metamorphosis in some tissues (Figure 3). Four GPCR genes, *Galr3* (encoding galanin receptor type 3), *GPCR4* (encoding the uncharacterized protein LOC110374861), *Opsin* (encoding red-sensitive opsin), and *Fzd4* (encoding frizzled 4) did not show increased expression during metamorphosis (Supplementary Figure 2). These results confirmed that the expression levels of nine GPCRs genes increased during metamorphosis, with or without tissue specificity, and might play roles in 20E-promoted metamorphosis.

### G Protein-Coupled Receptors Have Different Functions in 20E Pathway

**FIGURE 4:**
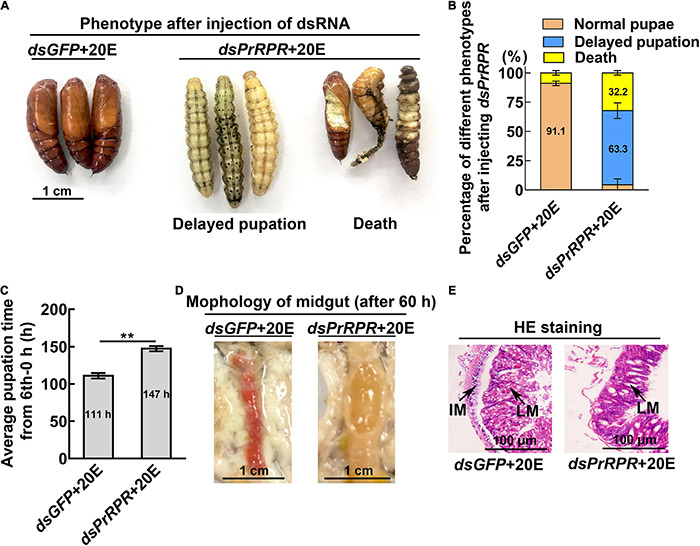
Knockdown of *PrRPR* delayed larval-pupal transition. **(A)** Phenotypes after *dsPrRPR* or *dsGFP* injection (sixth instar 6 h larvae for the first dsRNA injection, thrice at 24 h intervals, 1 μg dsRNA/larva), and 20E treatment (500 ng/larva). Images were obtained at 120 h after the first dsRNA injection. Scale bar = 1 cm. **(B)** Percentages of the phenotypes in **(A)**. **(C)** Statistical analysis of pupation time from 6th instar 0 h larvae developing to pupae. **(D)** Morphology of the midgut 60 h after the first dsRNA injection. **(E)** HE-stained midgut after knockdown of *PrRPR*, observed at 60 h after the first dsRNA injection. LM, larval midgut; IM, imaginal midgut. HE staining showing the morphology of the midgut. The bars represent 100 μm. The experiments were performed in triplicate, and significant differences were calculated using Student’s *t*-test (**p < 0.01). The bars indicate the mean ± SD.

Among the nine GPCRs that showed increased expression during metamorphosis, three GPCRs-*Fshr*, *Rya-R*, and *Npfr* were not successfully knocked down in larvae using RNA interference (RNAi). Other six GPCRs, *PrRPR*, *Smo*, *Akhr*, *Htr*, *Fzd7*, and *TkR86C*, which showed increased expression during metamorphosis in all tissues or in some tissues, were knocked down in larvae using RNAi to examine their roles in 20E-promoted earlier pupation. In the *dsGFP* plus 20E treatment group, 91.1% of the larvae pupated in 111 h (timed from the 6th instar 6 h to pupae). However, knockdown of *PrRPR* caused 63.3% of the larvae delayed pupation for 36 h, and increased death, compared with *dsGFP* plus 20E (Figures 4A–C). In addition, the midgut did not show a red color, a sign of programmed cell death (Wang et al., 2007; Hakim et al., 2010), in the *dsPrRPR* plus 20E treatment group compared with that in the *dsGFP* plus 20E control (Figure 4D). Hematoxylin and eosin (HE) staining showed that the imaginal midgut formed after *dsGFP* control injection, indicating the occurrence of midgut remodeling. In contrast, the imaginal midgut did not form after *dsPrRPR* injection for 60 h (Figure 4E). Similar results were obtained after knockdown of *Smo*, *Akhr*, and *Htr*. Compared with the *dsGFP* + 20E group, 53–65% of larvae delayed pupation for 24–43 h, and the midgut did not change to red or remodel on time (Supplementary Figures 3–5). These results suggested that these four GPCRs play roles in 20E-promoted pupation.

**FIGURE 5:**
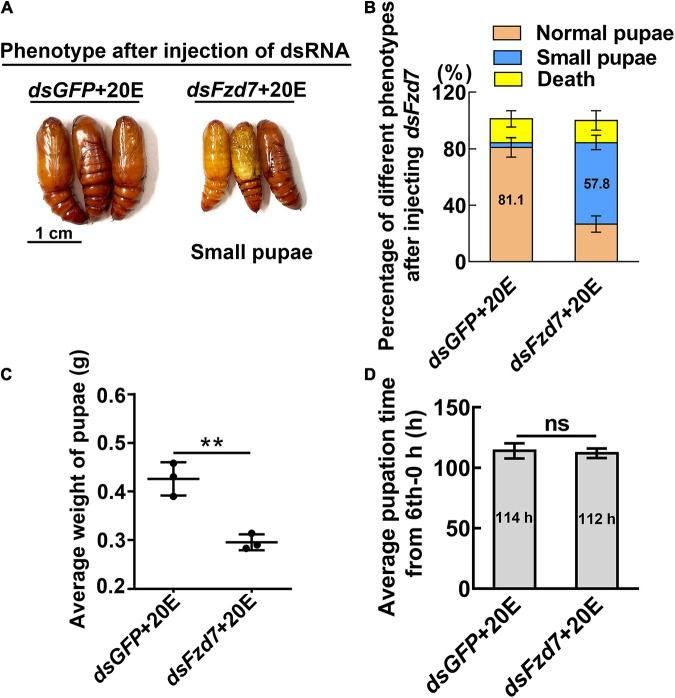
Knockdown of *Fzd7* decreased body weight. **(A)** Phenotypes after *dsFzd7* or *dsGFP* injection (sixth instar 6 h larvae for the first dsRNA injection, thrice at 24 h intervals, 1 μg dsRNA/larva), and 20E treatment (500 ng/larva). Images were obtained at 120 h after the first dsRNA injection. Scale bar = 1 cm. **(B)** Percentages of the phenotypes in **(A)**. **(C)** Statistical analysis of average weight of a pupa at day one, by individually weight, after *Fzd7* knockdown by injection with *dsFzd7*. **(D)** Statistical analysis of pupation time from 6th instar 0 h larvae developing to pupae. The experiments were performed in triplicate, and significant differences were calculated using Student’s *t*-test (**p < 0.01). The bars indicate the mean ± SD.

However, knockdown of *Fzd7* caused 57.8% of the larvae to form small pupae (Figures 5A,B). The pupal weight decreased to an average of 0.29 g compared with 0.43 g of the *dsGFP* injection control, with no significant difference in pupation time compared with the control group (Figures 5C,D). These results suggested that FZD7 is involved in larval growth. However, knockdown of *TkR86C* resulted in no abnormal phenotype (Supplementary Figure 6). The efficacy of RNAi was confirmed for these GPCRs, and except for *Smo* and *Fzd7*, which decreased after knockdown of *Htr*, no off target effects were detected for the other GPCRs (Supplementary Figure 7). These results showed that different GPCRs in the 20E signaling pathway play different roles in regulating growth and metamorphosis.

### 20E, via Different G Protein-Coupled Receptors, Regulates Gene Expression

**FIGURE 6:**
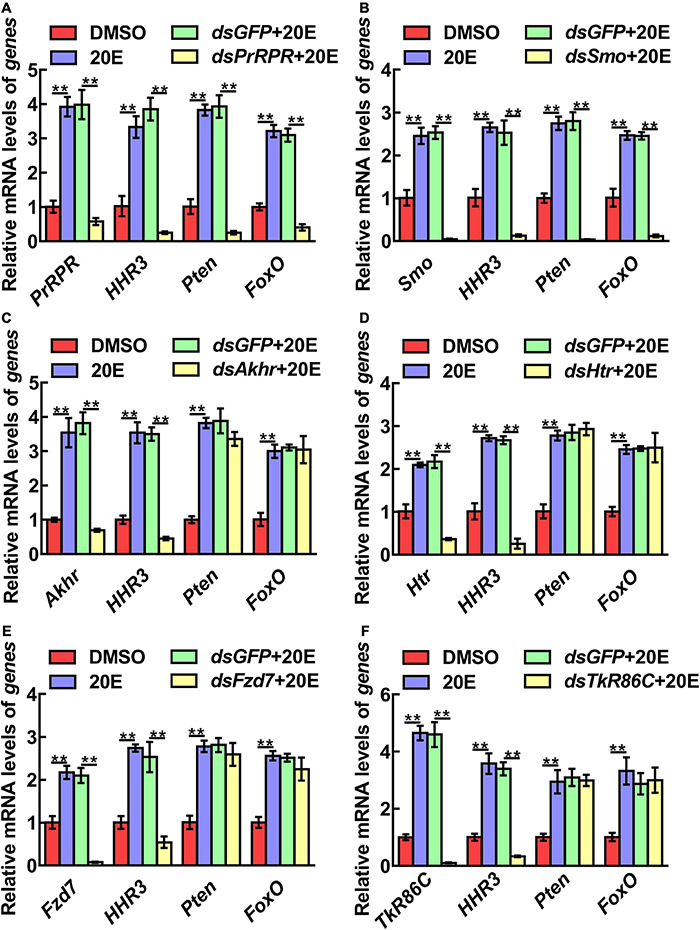
qRT-PCR showing the relative mRNA levels of genes after knockdown GPCRs. **(A–F)** *PrRPR*, *Smo*, *Akhr*, *Htr*, *Fzd7*, and *TkR86C* knockdown and transcript levels of *HHR3*, *Pten* and *FoxO* in 6th–72 h larval midgut (1 μg *dsRNA*/larva). DMSO or 20E (500 ng/larva) were added for 12 h. DMSO was used as the solvent control. All the experiments were performed in triplicate, and significant differences were calculated using Student’s *t*-test (**p < 0.01). The bars indicate the mean ± SD.

**FIGURE 7:**
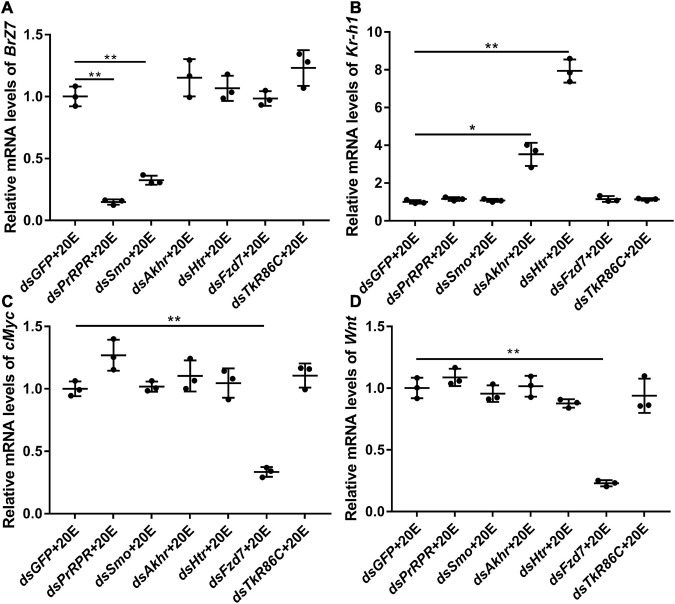
qRT-PCR showing the relative mRNA levels of *BrZ7, Kr-h1, cMyc*, and *Wnt* after knocking down GPCRs. **(A–D)** The transcript levels of *BrZ7*, *Kr-h1*, *cMyc*, and *Wnt* in the larval midgut after knockdown of GPCRs *PrRPR*, *Smo*, *Akhr*, *Htr*, *Fzd7*, and *TkR86C* [sixth instar 6 h larvae for the first dsRNA injection, thrice at 24 h intervals, 1 μg dsRNA/larva; 20E (500 ng/larva) for 12 h]. DMSO was used as the solvent control. All the experiments were performed in triplicate, and significant differences were calculated using Student’s *t*-test (*p < 0.05; **p < 0.01). The bars indicate the mean ± SD.

The mechanism by which knockdown of the six GPCRs caused different outcomes was addressed by examining gene expression, including *HHR3*, *Pten*, *FoxO*, and *BrZ7*, which play roles in 20E-induced metamorphosis, *Kr-h1*, which plays role in JH signaling, *Wnt* and *cMyc*, which play roles in growth. qRT-PCR analysis showed that the expression levels of the six GPCR genes were upregulated by 20E in the midgut, confirming their responses to 20E induction. After knockdown of the six GPCR genes by RNAi in larvae, the mRNA levels of *HHR3* decreased, suggesting that these six GPCRs play roles in 20E signaling. However, the expression of *Pten* and *FoxO* decreased only after *PrRPR* and *Smo* knockdown (Figures 6A,B), but not after *Akhr*, *Htr*, *Fzd7*, and *TkR86C* knockdown (Figures 6C–F). Furthermore, *BrZ7* expression decreased after knockdown of *PrRPR* and *Smo*, *Kr-h1* expression increased after *Akhr* and *Htr* knockdown, and *Wnt* and *cMyc* decreased after knockdown of *Fzd7* (Figure 7). These results revealed that 20E, via different GPCRs, integrates insect pupation and growth by regulating the expression of various genes.

To support this conclusion, the previous reported GPCRs that transmit the 20E signal, ErGPCR-1, ErGPCR-2, ErGPCR-3, and DopEcR, were examined for their regulation of gene expression. The results showed that the expression of *HHR3* decreased after knockdown of *ErGPCR-1, ErGPCR-2*, *ErGPCR-3*, and *DopEcR*; however, *Pten* and *FoxO* expression decreased after *ErGPCR-1* knockdown, but not after *ErGPCR-2*, *ErGPCR-3*, or *DopEcR* knockdown (Supplementary Figure 8A), which confirmed that different GPCRs regulate the expression of different genes in the 20E pathway. The RNA interference efficiency of these four GPCRs was confirmed (Supplementary Figures 8B–E).

### Prolactin-Releasing Peptide Receptor Binds 20E

**FIGURE 8:**
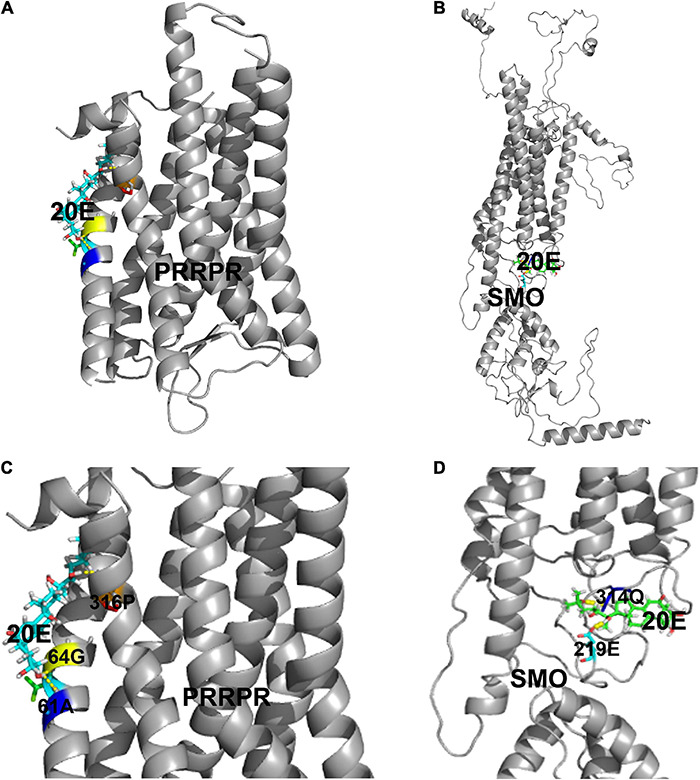
Modeling of the ligand-binding complex of PRRPR and SMO. Prediction from the Surflex-Dock (SFXC) program from the SYBYL X2.0 software. **(A,B)** Whole structures of PRRPR and docked 20E, and SMO and docked 20E, respectively. **(C,D)** A closer view of the docking model pockets of PRRPR-20E and SMO-20E complexes. The amino acid residues with which 20E can form hydrogen bonds were shown in the figure.

To identify new GPCR functioning as cell membrane receptor of 20E, PRRPR and SMO were further analyzed for their binding 20E to determine their receptor roles in 20E signaling. Surflex-Dock (SFXC) in SYBYL X2.0 software (Certara, Princeton, NJ, United States) was used to dock 20E to PRRPR and SMO to predict the possibility of PRRPR and SMO binding 20E (Figures 8A,B). 20E forms hydrogen bonds with Ala-61, Gly-64, and Pro-316, of PRRPR, and Gln-314 and Glu-219 of SMO (Figures 8C,D). The scores for PRRPR and SMO binding to 20E were 2.96, and –0.78, respectively. These data predicted that PRRPR has a higher binding ability to 20E than SMO.

**FIGURE 9:**
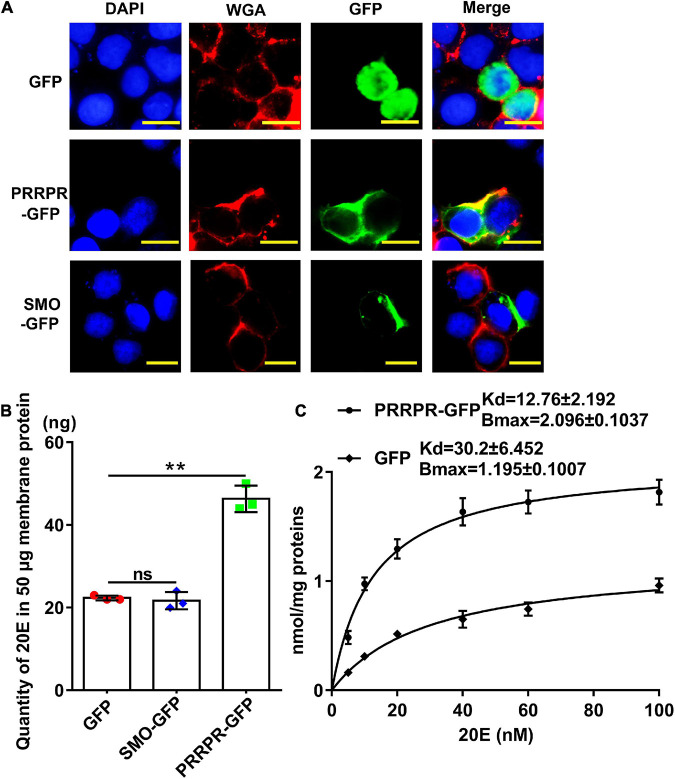
Detection of 20E that was bound by the cell membrane proteins from HaEpi cells overexpressing GPCRs. **(A)** Cell membrane localization of overexpressed GFP, PRRPR, and SMO. Blue: Nuclei stained with DAPI. Red: Cell membrane was marked by WGA. Green: Fluorescence from GFP and various GPCRs fused with GFP. Scale bar = 20 μm. **(B)** Quantity of 20E bound by 50 μg of membrane proteins from HaEpi cells overexpressing GFP, PRRPR-GFP, or SMO-GFP. **(C)** Saturation binding curves of HaEpi cell membranes from cells overexpressing GFP, PRRPR-GFP, SMO-GFP to 20E. Error bars represent the SD of three replicates. Asterisks indicate significant differences according to Student’s *t*-tests (**p < 0.01).

PRRPR-GFP and SMO-GFP were overexpressed in an *H. armigera* epidermal cell line (HaEpi) to address their binding to 20E. Green fluorescent protein (GFP) was overexpressed as a tag control. The overexpressed PRRPR-GFP and SMO-GFP were confirmed to be located in the cell membrane (Figure 9A). A binding assay using a 20-hydroxyecdysone enzyme immunoassay (20E-EIA) showed that the amount of 20E bound by the cell membrane from the PRRPR-GFP-overexpressing cells increased significantly compared with that bound by the GFP-overexpressing cells. However, the amount of 20E bound by cell membranes from SMO-GFP overexpressing cells did not increase compared with that of the GFP-overexpressing cells (Figure 9B). These results suggested that PRRPR could bind 20E in the cell membrane.

A saturation-binding curve was constructed using 20E-EIA to further examine the affinity of GPCRs to 20E by calculating their dissociation constants (Kds). The saturable specific binding of cell membranes from cells overexpressing PRRPR-GFP to 20E had a Bmax of 2.096 ± 0.1037 nmol/mg protein and a Kd of 12.76 ± 2.192 nM. In comparison, the saturation binding of cell membranes from cells overexpressing GFP to 20E had a Bmax of 1.195 ± 0.1007 nmol/mg protein and a Kd of 30.2 ± 6.452 nM (cells overexpressing GFP still have other GPCRs on their cell membranes) (Figure 9C). The 20E-EIA assay is based on competition between the unlabeled 20E (20E bound to GPCR) and acetyl choline esterase (AChE)-labeled 20E (Tracer) for the limited-specific rabbit anti-20E antiserum; therefore, an inhibition or competitive curve was not detected. These data confirmed that the PRRPR-GFP could bind 20E.

## Discussion

This research identified and classified all classical GPCRs in the *H. armigera* genome. The GPCRs that function as 20E receptors were classified in classes A, B and class C or F. Further study revealed that different GPCRs showed different expression profiles and mediated the expression of different genes in 20E signaling. PRRPR was determined as a new GPCR cell membrane receptor. These data explained the mechanism by which several GPCRs are involved in the signaling of the same ligand.

### Identification and Classification of G Protein-Coupled Receptors in *Helicoverpa armigera* Genome

We identified 122 genes encoding classical GPCRs in the *H. armigera* genome. The GPCRs were classified into categories A, B, C, or F, which was relatively consistent with the classification of GPCRs in *Drosophila* (Hanlon and Andrew, 2015). Most of the GPCRs were classified consistently with their classification in the genome; however, some GPCRs were mixed in different classes in our study when using the full open reading frames (ORFs). We found 19 Mth-like GPCRs in the *H. armigera* genome, which is close to the 16 Mth-like GPCRs in *D. melanogaster* (Patel et al., 2012), but more than the 7 found in *Anopheles*, and the 2 found in *B. mori* (Fan et al., 2010). The Mth-like GPCRs play various roles in regulating the metabolism, aging, and self-balance to high temperature, hunger, dryness, and oxidative damage (Friedrich and Jones, 2016). In insects, Mth-like GPCRs are known to play roles in the setting of the endogenous circadian clocks (Mertens et al., 2007), regulation of fluid and ion secretion (Reagan, 1994), as well as the stress response and longevity (Lin et al., 1998).

### 20E, via Different G Protein-Coupled Receptors, Regulates Gene Expression

The involvement of several GPCRs in a same signal, such as 20E signaling, is an intriguing phenomenon. The differences among the GPCRs in 20E signaling have been explained by their induced downstream effects, including ErGPCR-1 inducing the Ca^2+^-PKC signaling, while ErGPCR-2 inducing the GPCR-cAMP-PKA and GPCR-Ca^2+^-PKC signaling, increasing 20E entry, and being internalized by 20E induction. DopEcR of *H. armigera* directly interacts with Gαs and Gαq under the induction of 20E to increase the levels of cAMP and Ca^2+^ (Zhao, 2020). ErGPCR-3 has very similar characteristics to ErGPCR-2 (Kang et al., 2021). Different GPCRs can cross react with different G proteins (Flock et al., 2017). Here, we further revealed that GPCRs have quite different expression profiles in tissues and at different developmental stages. Importantly, 20E, via different GPCRs, regulates the expression of various genes, including via PRRPR and SMO, which upregulate the expression of *Pten*, *FoxO*, and *BrZ7* to promote pupation. 20E, via AKHR and HTR, represses the expression of *Kr-h1* to promote pupation. 20E via FZD7 upregulates the expression of *Wnt* and *cMyc* to promote growth. 20E signaling also promotes wing disk development (Mirth et al., 2009). By the integration and competition of different signals induced by different ligands *in vivo*, 20E regulates pupation. These findings in 20E signaling first revealed the mechanism by which several GPCRs transmit the same signal to regulate the expression of different genes in the network of the cells. In this work, we performed the screen based on the expression levels of GPCRs. There might be GPCRs transmit external signals in an expression-independent manner, which needs further study.

Our results suggested PRRPR, SMO, AKHR, HTR, FZD7, and TKR86C are involved in 20E-inducing *HHR3* expression, suggesting that they transmit 20E signals. In humans, PRRPR is a neuropeptide prolactin receptor (Dodd and Luckman, 2013). Human SMO participates in hedgehog signaling to guide cell differentiation, proliferation, and survival (Wu et al., 2017). FZD7 is the most important WNT receptor involved in cancer development and progression in mammals (King et al., 2012). In insects, AKHR binds AKH (adipokinetic hormone) to increase lipolysis, glycogenolysis, and trehalose production (Van der Horst et al., 2001; Bednarova et al., 2013; Baumbach et al., 2014). HTR (5-HT receptor) plays a key role in morphogenesis in the insect nervous system (Blenau and Thamm, 2011). TkR86C is the neurokinin K receptor in *D. melanogaster* that plays a role in neuromodulation in the central nervous system, participating in the processing of sensory information and the control of motor activities (Vanden Broeck et al., 1999). The role of TkR86C in insect needs further study. Here, we revealed a new function of these GPCRs in 20E signaling.

### G Protein-Coupled Receptors Can Transmit 20E Signals Whether They Bind 20E or Not

It has been suggested that cells or cell membranes that overexpress GPCRs can bind steroid hormones in *Drosophila* (Srivastava et al., 2005) and mammals (Maggiolini and Picard, 2010). We found that PRRPR could bind 20E with the saturable specific binding Kd of 12.76 ± 2.192 nM. However, SMO could not bind 20E, although SMO transmits the 20E signal and is involved in 20E-induced pupation. In our previous study, we found that ErGPCR-1, ErGPCR-2, ErGPCR-3, and DopEcR can transmit 20E signals in *H. armigera*. ErGPCR-2, ErGPCR-3, and DopEcR can bind 20E, but ErGPCR-1 cannot (Kang et al., 2019, 2021). These data suggested that GPCRs transmit 20E signal with or without binding 20E. This might be because GPCRs loosely or dynamically bind their ligands (Nygaard et al., 2013; Strasser et al., 2017). Another possibility is that 20E competes with another ligand, such as dopamine, in *H. armigera* (Kang et al., 2019). GPCRs might also play roles in other pathways after upregulation by 20E, which requires further study.

G protein-coupled receptors share a seven transmembrane domain structural architecture (Latorraca et al., 2017). Except ErGPCR-1, ErGPCR-2, ErGPCR-3, and DopEcR have been reported to bind 20E. In addition to DopEcR in class A, ErGPCR-1, ErGPCR-2, and ErGPCR-3 belong to the Mth-like GPCRs in class B. An important feature of Mth-like GPCRs is the presence of 10 cysteine residues that form five disulfide bonds (West et al., 2001). The long N-terminal domains tend to recognize peptide ligands, such as hormones and neuropeptides (Cardoso et al., 2005, 2010), such as secretin; therefore, these GPCRs are also called secretin receptors (Cvejic et al., 2004; Ja et al., 2009). The relationship between the structure of GPCR and its binding 20E is unclear now. Up to date, several GPCRs can bind 20E, therefore, the upregulation of GPCR expression by 20E is likely a key factor to perform their functions. The mechanism that 20E upregulates GPCR expression differentially needs further studied. Mth-like GPCRs are not present in vertebrates, but are more abundant in arthropods (Patel et al., 2012; Araujo et al., 2013; de Mendoza et al., 2016), and thus represent targets for insecticides.

## Conclusion

**FIGURE 10:**
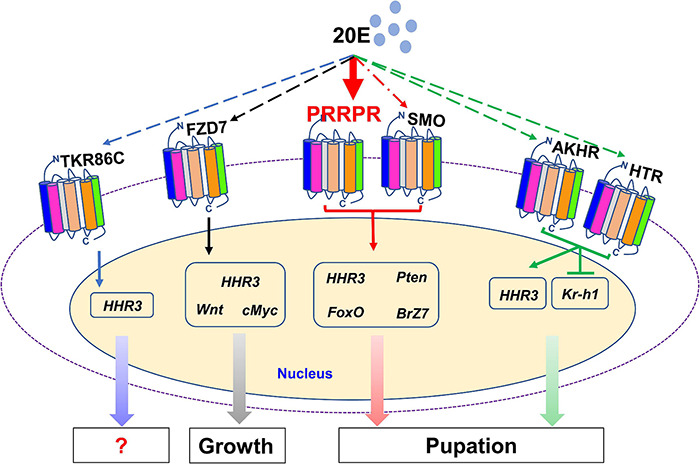
20E, through different GPCRs, regulates the expression of different genes to integrate growth and pupation. 20E through PRRPR, SMO, AKHR, HTR, FZD7, and TKR86C, regulates the expression of *HHR3*. 20E via PRRPR and SMO upregulates the expression of *Pten*, *FoxO*, and *BrZ7* to promote pupation. 20E, via AKHR and HTR, represses the expression of *Kr-h1* to promote pupation. 20E, via FZD7, upregulates the expression of *Wnt* and *cMyc* for growth.

There are 122 classical GPCRs in the genome of *H*. *armigera*. The GPCRs that transmit 20E signal were classified in classes A, B and class C or F. Various GPCRs transmit 20E signals according to their different expression patterns in tissues and their increased expression during metamorphosis. 20E, via different GPCRs, regulates the expression of various genes, thus promoting pupation by integrating different signals *in vivo*. PRRPR binds 20E and is a newly identified 20E cell membrane receptor (Figure 10).

## Materials and Methods

### Identification of G Protein-Coupled Receptors

Putative *H. armigera* GPCRs were identified in four steps: First, we downloaded all the GPCR protein sequences of *Drosophila* (Hanlon and Andrew, 2015) and *B. mori* (Fan et al., 2010). *B. mori* protein sequences were retrieved from the NCBI sequence repository^2^. We obtained 90 classical GPCRs from *B. mori*. *Drosophila* protein sequences were retrieved from FlyBase^3^ and the NCBI database. We obtained 116 classical *Drosophila* GPCRs. Second, preliminary screening to obtain putative GPCRs of *H. armigera* was performed using NCBI BLAST based on downloaded *Drosophila* and *B. mori* GPCRs. We queried the *H. armigera* proteome^4^ using each GPCR sequence from *Drosophila* and *B*. *mori* separately and selected the protein sequences with the highest scores. Third, the protein sequences with highest score were then used as query sequences one by one in a BLAST search against the proteome from *H. armigera* to obtain other GPCRs sequences that were not found in the previous step. Finally, we removed the repetitive sequences in the protein sequence obtained in the above steps. Then, NCBI conserved domain search service (CD search)^5^ and SMART online software^6^ were used to predict the structure of these protein sequences, and the GPCRs were seven-transmembrane domain proteins (7TMPs) were obtained.

GPCRs were identified from the transcriptomes of 6th–24 h larvae and 6th–72 h larvae. The transcriptomes were analyzed once without technique replicates. However, the samples were collected from several larvae to normalize the individual differences. The mRNA levels of GPCRs were examined after injection of 20E or Double-Stranded RNA (dsRNA) using qRT-PCR, with an equal amount of diluted DMSO as a solvent control.

### Phylogenetics Analyses

The classification of *Drosophila* and *Homo sapiens* proteins in each GPCR family is clear and detailed. We classified the potential GPCRs in the *H. armigera* genome into various categories based on sequence homology. *Drosophila* and *Homo sapiens* GPCRs were used as guides, and the MEGA 6.0 software was used to construct phylogenetic trees using the Neighbor-Joining method with 1000 bootstraps (Tamura et al., 2013).

### Insects

*Helicoverpa armigera* larvae were raised in the insect culture room at 25–27°C under a photoperiod of 14 h light/10 h dark. The larvae are reared on a previously described artificial diet (Zhao et al., 1998).

### Quantitative Real-Time Reverse Transcription PCR

The total RNA was extracted using the Trizol reagent (TransGen Biotech, Beijing, China). cDNA was synthesized from the total RNA using a FastQuant RT Kit (Tiangen Biotech, Beijing, China). qRT-PCR was then carried out using the cDNA as the template in a CFX96 real-time system (Bio-Rad, Hercules, CA, United States) with 2 × SYBR qRT-PCR pre-mixture (TransGen Biotech). All the primers used are listed in Supplementary Table 2. *Actb* encodes a type of actin, which is a structural component of the cytoskeleton microfilaments. The *Actb* gene is highly conserved and highly expressed at the mRNA level (Butet et al., 2014). In many studies, including studies on different developmental stages and different tissues in *H. armigera* (Di et al., 2020), *Actb* is considered a suitable internal reference gene (Lu et al., 2013; Gao et al., 2017). *H. armigera Actb* (encoding beta actin; GenBank accession no. EU52707) was used as the internal standard. All data were from at least three biological replicates and were analyzed using the 2^−ΔΔ*CT*^ method (ΔΔCT = ΔCT_treated sample_-ΔCT_control_, ΔCT = CT_gene_-CT*_Actb_*) (Livak and Schmittgen, 2001).

### 20E Induction in Larvae

The 20E powder (10 mg) was dissolved in 1 mL DMSO as a storage solution and diluted with phosphate-buffered saline (PBS; 140 mM NaCl, 10 mM sodium phosphate, pH 7.4) for experiment. 20E was injected into the hemocoel from the side of the larval abdomen. The control groups were treated with the equal amount of diluted DMSO.

### Double-Stranded RNA Synthesis

RNA interference (RNAi) has been used for gene knockdown in many moths (Xu et al., 2016). The long dsRNA is broken down into smaller fragments *in vivo* (Zamore et al., 2000) and specifically and successfully inhibits the expression of target genes in worms (Fire et al., 1998). DNA fragments– 5′-583 bp-1037 bp-3′ of *Fzd7*, 5′-586 bp-1109 bp-3′ of *Htr*, 5′-43 bp-586 bp-3′ of *PrRPR*, 5′-1195 bp-1858 bp-3′ of *Smo*, 5′-641 bp-1045 bp-3′ of *Akhr*, 5′-65 bp-740 bp-3′ of *TkR86C*– were amplified as the template for dsRNA synthesis using the primers RNAiF and RNAiR. A T7 promoter sequence was added to the RNAi primers (Supplementary Table 2). The cDNA of the target gene was amplified using a single PCR reaction and was used as the template to synthesize dsRNA. The dsRNA was synthesized using MEGAscript RNAi kit (Ambion, Austin, TX, United States) according to the instruction manual. Next, the product was purified using the phenol-chloroform method. The quality of the synthesized dsRNA was quantified using a micro-spectrophotometer and detected using 1% agarose gel electrophoresis.

### RNA Interference of Genes in Larvae

The dsRNA was diluted with PBS. The sixth instar 6 h larvae were placed on ice for 15 min until they did not move. A sterile micro syringe was used to inject 1 μg of dsRNA into the hemocoel from the side of the larval abdomen (taking care not to touch the midgut). dsRNAs were injected three times at 24 h intervals. The control groups were treated with the same amount of *dsGFP*. Each experimental group and control group contained 30 larvae and three independent biological replicates were performed. Total RNA was extracted using Trizol reagent (TransGen Biotech) and qRT-PCR was performed to detect the effects of RNAi at 24 h after the last injection.

### Hematoxylin-Eosin Staining

The midgut dissected from the larva was washed with PBS, and then fixed in 4% paraformaldehyde at 4°C overnight. The fixed tissue was submitted to a professional company (Servicebio, Wuhan, China) for processing into glass slides and for Hematoxylin-Eosin (HE) staining.

### Overexpression of Prolactin-Releasing Peptide Receptor and Smoothened in HaEpi Cells

The pIEx-4-GFP-His vector that was fused with a sequence encoding the green fluorescent protein (GFP) and used for experiments in the insect cell line. The open reading frames (ORFs) of *PrRPR* (GenBank accession no. XP_021184170.1) and *Smo* (GenBank accession no. XP_021189185.1) were amplified using primers (Supplementary Table 1) and inserted into the vector. Then, 5 μg of the recombinant plasmids were transfected into HaEpi cells using the QuickShuttle-enhanced transfection reagent (Biodragon Immunotech, Beijing, China). After 48 h of transfection, further experiments were conducted.

### Immunocytochemistry

After PRRPR-GFP and SMO-GFP were overexpressed for 48 h, HaEpi cells were washed three times with 500 μL of Dulbecco’s phosphate-buffered saline (DPBS; 137 mM NaCl, 2.7 mM KCl, 1.5 mM KH_2_PO_4_ and 8 mM Na_2_HPO_4_, pH 7.4), and fixed with PBS containing 4% paraformaldehyde for 10 min in the dark at room temperature. The fixed cells were washed three times for 3 min each. The plasma membrane was stained using wheat germ agglutinin (WGA; Sigma-Aldrich, St. Louis, MO, United States; 1 μg/mL in PBS) in the dark for 4 min and then washed with PBS six times. Nuclei were stained with 4′-6-diamidino-2-phenylindole dihydrochloride (DAPI; Sangon Biotech, Shanghai, China; 1 μg/mL in PBS) in the dark at room temperature for 10 min and then washed with PBS six times. Fluorescence was detected using an Olympus BX51 fluorescence microscope (Olympus, Tokyo, Japan). The negative control (GFP expression) was treated following the same method.

### 20-Hydroxyecdysone Enzyme Immunoassay

The 20-hydroxyecdysone enzyme immunoassay (20E-EIA) is based on the competition between unlabeled 20E (free 20E) and acetylcholinesterase (AChE)-labeled 20E (Tracer) for limited specific rabbit anti-20E antiserum. The rabbit anti-20E antiserum was combined with the mouse anti-rabbit monoclonal antibody coated-plate. Then, the plate was washed using the wash buffer included with the 20-Hydroxyecdysone Enzyme Immunoassay kit (20E-EIA kit) (Bertin Pharma, Paris, France) (2 mL of concentrated Wash Buffer #A17000 was diluted by 800 mL of UltraPure water then added 400 μL of Tween20 #A12000) to remove all unbound reagents. Then, tracer and free 20E in samples were added into the wells and the plates were incubated at 4°C overnight. After washing the plate five times with wash buffer, 200 μL Ellman’s reagent (an enzymatic substrate for AChE and a chromogen) was added to the wells, and the plate was then incubated with an orbital shaker at 400 rpm in the dark at room temperature. AChE-labeled 20E acts on the substrate in Ellman’s Reagent to form a yellow compound, which can strongly absorb light at 414 nm. The intensity of the color was detected using a spectrophotometer (Infinite M200PRO NanoQuant, Tecan, Grödig, Austria) at 414 nm. The optical density was proportional to the amount of tracer bound to the well and inversely proportional to the amount of 20E in the sample. The 20E standard curve generated by this method was used to determine the quantity of 20E bound to cell membrane proteins.

### Detection of the 20E Quantity Bound by the Cell Membranes of HaEpi Cells

PRRPR-GFP and SMO-GFP were overexpressed in HaEpi cells in a 25 cm^2^ cell culture bottle, respectively. After washing with DPBS twice, the cells were incubated in Grace’s medium containing 1 μM 20E for 5 min at 27°C to allow 20E to bind to the cell membrane. The cells were then collected by centrifugation at 1,700 × *g* at 4°C for 5 min and the pellet was resuspended in 500 μL enzyme immunoassay (EIA) buffer (Bertin Pharma, Paris, France) and sonicated for 5 min. The pelleted cell membrane debris was resuspended in 100 μL EIA buffer after centrifugation at 4°C at 48,000 × *g* for 1 h. Then, 50 μg of cell membrane proteins with fixed 20E in 50 μL EIA buffer was added with 450 μL EIA buffer and used to quantify 20E. The 20E-EIA kit was used to detect cell membrane bound-20E according to the manufacturer’s instructions.

### Statistical Analysis

Two-group datasets were analyzed using Student’s *t*-test and in the figures, an asterisk represents a significant difference (*p* < 0.05) and two asterisks represent an extremely significant difference (*p* < 0.01). Analysis of variance (ANOVA) was used for multiple comparisons and in the figures, different lowercase letters indicate significant differences (*p* < 0.05), and the bars indicate the mean ± standard deviation (SD) of three biological replicates. The details are provided in the figure legends.

## Data Availability Statement

The datasets presented in this study can be found in online repositories. The names of the repository/repositories and accession number(s) can be found in the article/Supplementary Material.

## Author Contributions

Y-LL and X-FZ designed the experiments and wrote the manuscript. D-JD, J-XW, and X-FZ conceived the idea. Y-LL, Y-XL, X-PW, X-LK, and K-QG performed the experiments. Y-LL and Y-XL performed the data analyses. All the authors contributed to the article and approved the submitted version.

## Conflict of Interest

The authors declare that the research was conducted in the absence of any commercial or financial relationships that could be construed as a potential conflict of interest.

## Publisher’s Note

All claims expressed in this article are solely those of the authors and do not necessarily represent those of their affiliated organizations, or those of the publisher, the editors and the reviewers. Any product that may be evaluated in this article, or claim that may be made by its manufacturer, is not guaranteed or endorsed by the publisher.

## Footnotes

1
https://www.ncbi.nlm.nih.gov/Taxonomy/Browser/wwwtax.cgi?mode=Info&id=29058

2
http://www.ncbi.nlm.nih.gov/

3
http://www.flybase.org/

4
https://www.ncbi.nlm.nih.gov/genome/?term=Helicoverpa+armigera

5
https://www.ncbi.nlm.nih.gov/Structure/cdd/wrpsb.cgi

6
http://smart.embl-heidelberg.de/smart/set_mode.cgi?NORMAL=1
