# Supplementary material for: Identification and Functional Analysis of G Protein-Coupled Receptors in 20-Hydroxyecdysone Signaling From the Helicoverpa armigera Genome
Source: Front Cell Dev Biol. 2021 Oct 26;9:753787. doi: 10.3389/fcell.2021.753787 (PMC8576438; doi:10.3389/fcell.2021.753787)
Supplement: Supplementary file 1 [file Data_Sheet_1.docx]

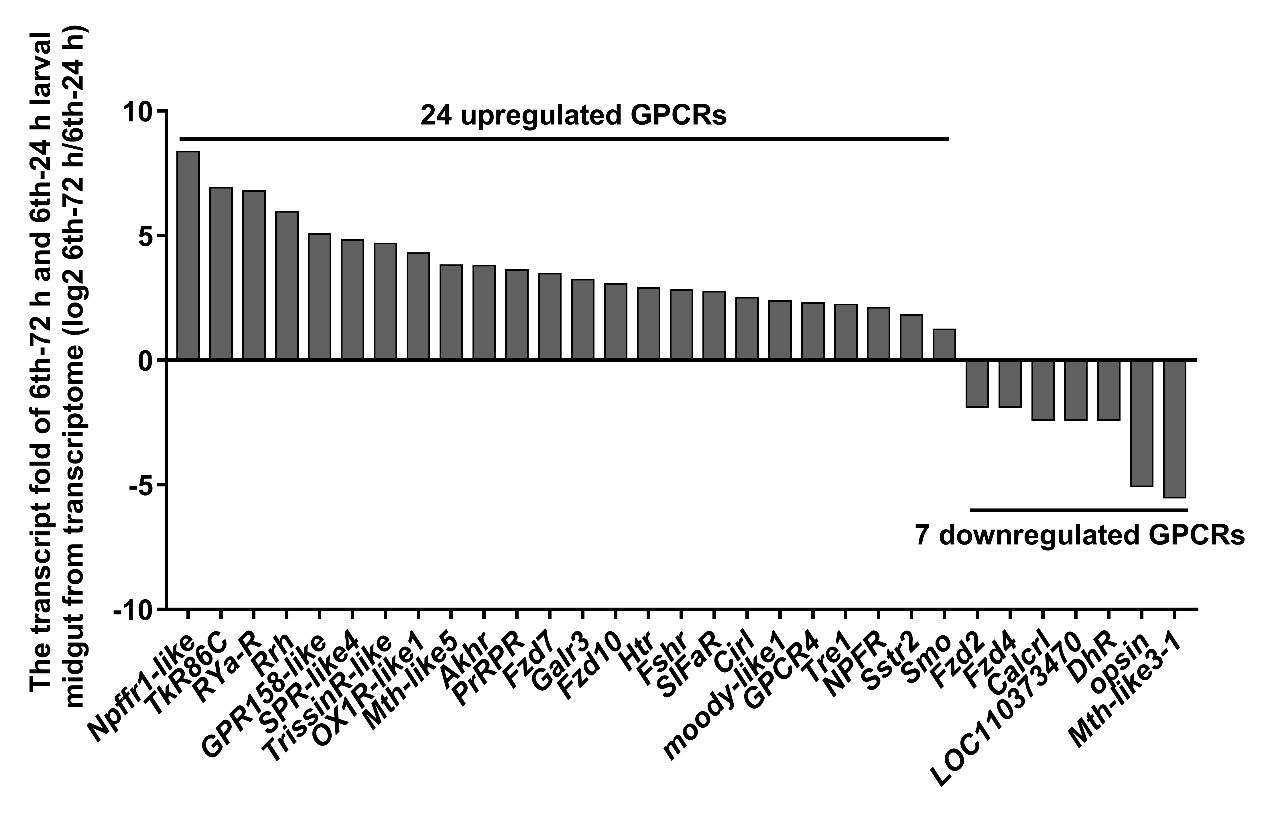


Supplementary Figure 1. The fold of GPCRs transcripts from the transcriptome of 6th–24 h larvae and 6th–72 h larvae. The ordinate indicates log2 (6th–72 h/6th–24 h). Npffr1-like (XP_021190007.1), TkR86C (XP_021196600.1), RYa-R (XP_021201003.1), Rrh (XP_021185923.1), GPR158-like (XP_021190148.1), SPR-like4 (XP_021190551.1), TrissinR-like (XP_021196001.1), OX1R-like1 (XP_021190844.1), Mth-like5 (XP_021194305.1), Akhr (XP_021200810.1), PrRPR (XP_021184170.1), Fzd7 (XP_021189247.1), Galr3 (XP_021192151.1), Fzd10 (XP_021199754.1), Htr (XP_021189580.1), Fshr (XP_021199987.1), SIFaR (XP_021193313.1), Cirl (XP_021184781.1), moody-like1 (XP_021201565.1), GPCR4 (XP_021188434.1), Tre1 (XP_021189408.1), NPFR (XP_021189907.1), Sstr2 (XP_021194801.1), Smo (XP_021189185.1), Fzd2 (XP_021189976.1), Fzd4 (XP_021192380.1), Calcrl (XP_021188050.1), LOC110373470 (XP_021186425.1), DhR (XP_021182085.1), opsin (XP_021189735.1), Mth-like3-1 (XP_021199338.1).


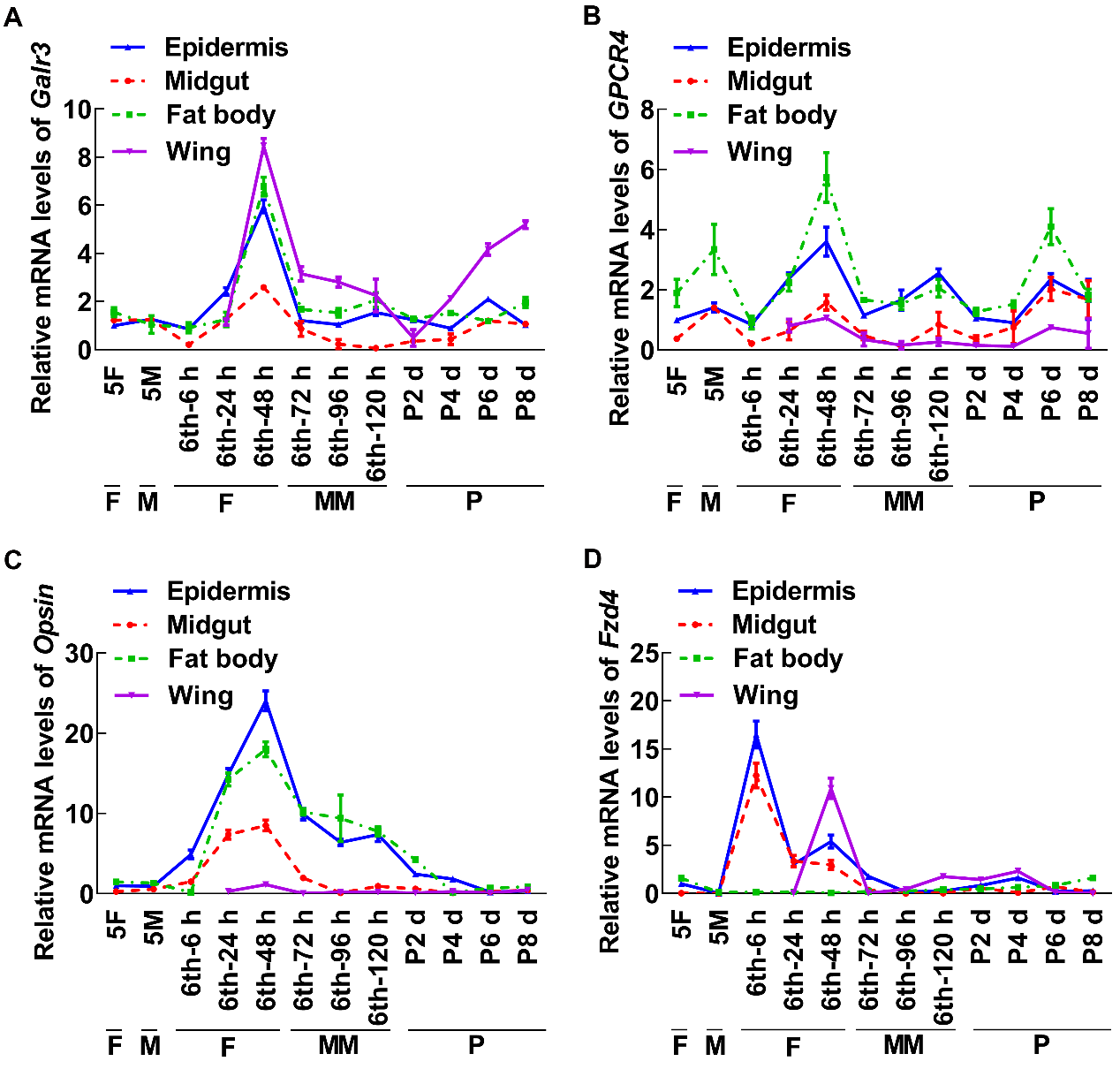


Supplementary Figure 2. qRT-PCR showing the expression profile of GPCRs that are not upregulated during metamorphosis. **(A-D)** The relative mRNA levels of *GalR3*, *GPCR4*, *Opsin*, and *Fzd4*. *Actb* was used as the control. All the experiments were performed in triplicate and the bars indicate mean ± SD.


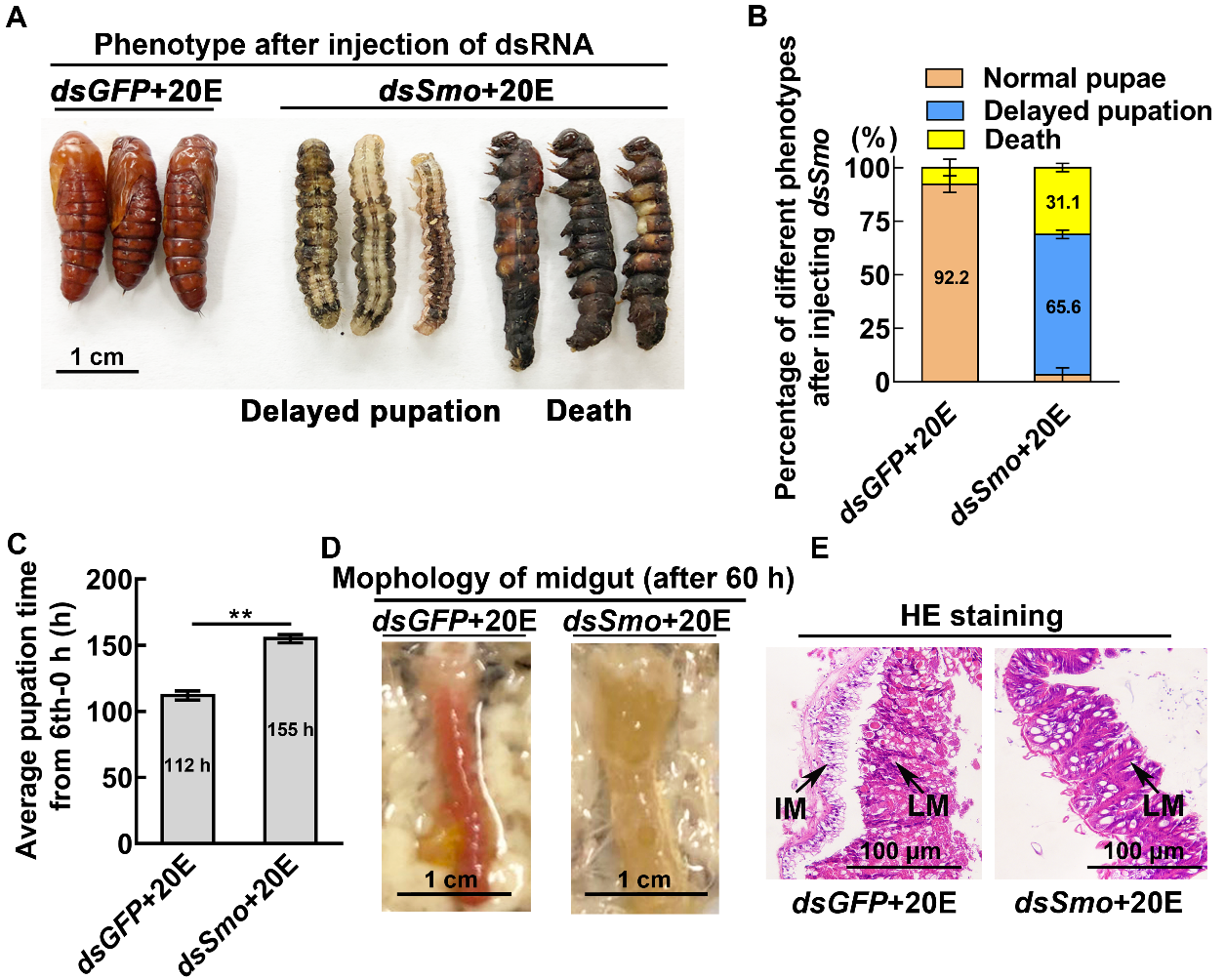


Supplementary Figure 3. Knockdown of *Smo* delayed larval-pupal transition. **(A)** Phenotypes after *dsSmo* or *dsGFP* injection (sixth instar 6 h larvae for the first dsRNA injection, thrice at 24 h intervals, 1 µg dsRNA/larva), and treated with 20E (500 ng/larva). Images were obtained at 120 h after the first dsRNA injection. Scale bar = 1 cm. **(B)** Percentages of the phenotypes in A. **(C)** Statistical analysis of pupation time from 6th instar 0 h larvae developing to pupae. **(D)** Morphology of the midgut 60 h after the first dsRNA injection. **(E)** HE-stained midgut after knockdown of *Smo*, observed at 60 h after the first dsRNA injection. The bars represent 100 μm. The experiments were performed in triplicate, and significant differences were calculated using Student’s *t*-test (***p* < 0.01). The bars indicate the mean ± SD.


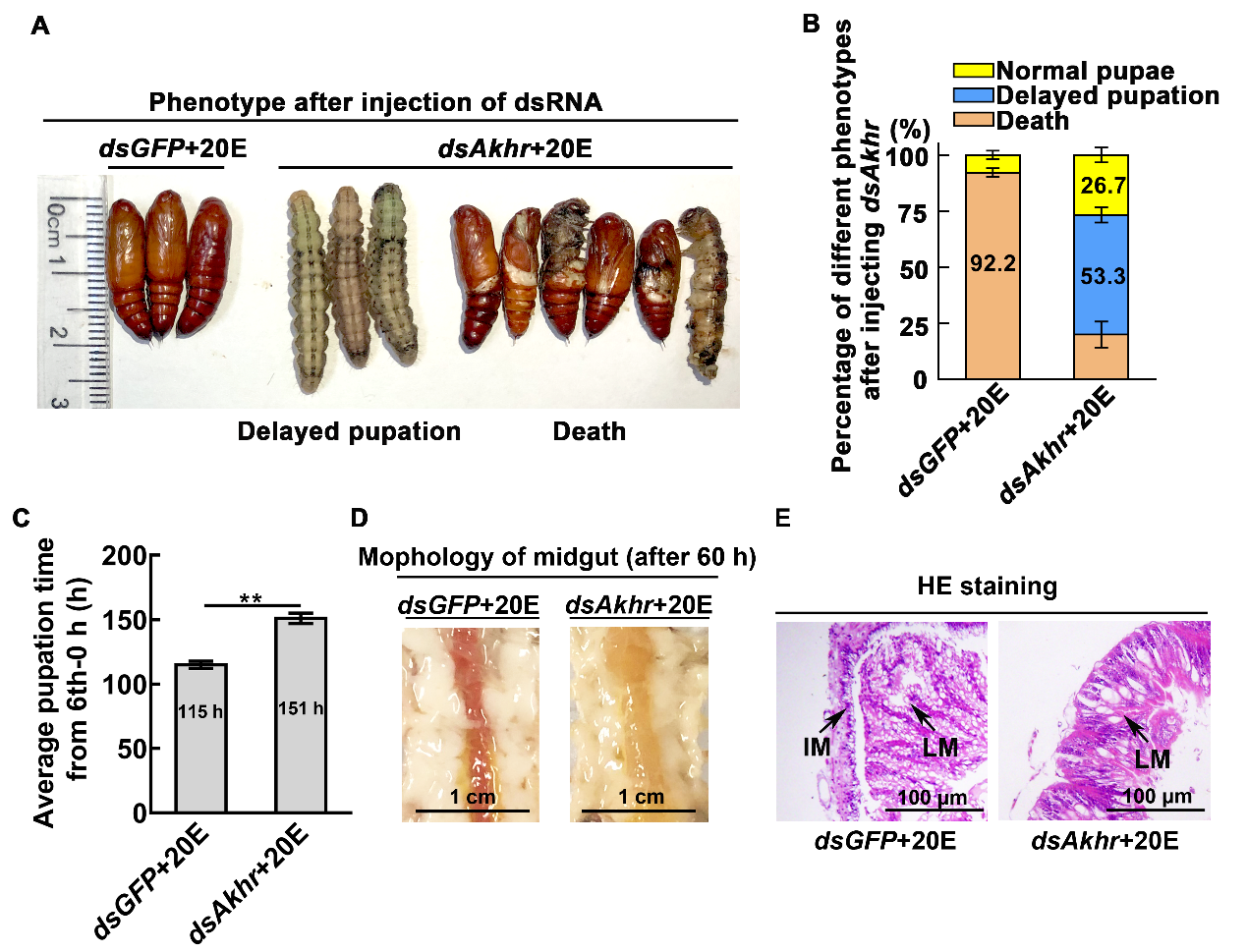


Supplementary Figure 4. Knockdown of *Akhr* delayed larval-pupal transition. (**A)** Phenotypes after *dsAkhr* or *dsGFP* injection (sixth instar 6 h larvae for the first dsRNA injection, thrice at 24 h intervals, 1 µg dsRNA/larva), and treated with 20E (500 ng/larva). Images were obtained at 120 h after the first dsRNA injection. **(B)** Percentages of the phenotypes in A. **(C)** Statistical analysis of pupation time from 6th instar 0 h larvae developing to pupae. **(D)** Morphology of the midgut 60 h after the first dsRNA injection. **(E)** HE-stained midgut after knockdown of *Akhr*, observed at 60 h after the first dsRNA injection. The bars represent 100 μm. The experiments were performed in triplicate, and significant differences were calculated using Student’s *t*-test (***p* < 0.01). The bars indicate the mean ± SD.


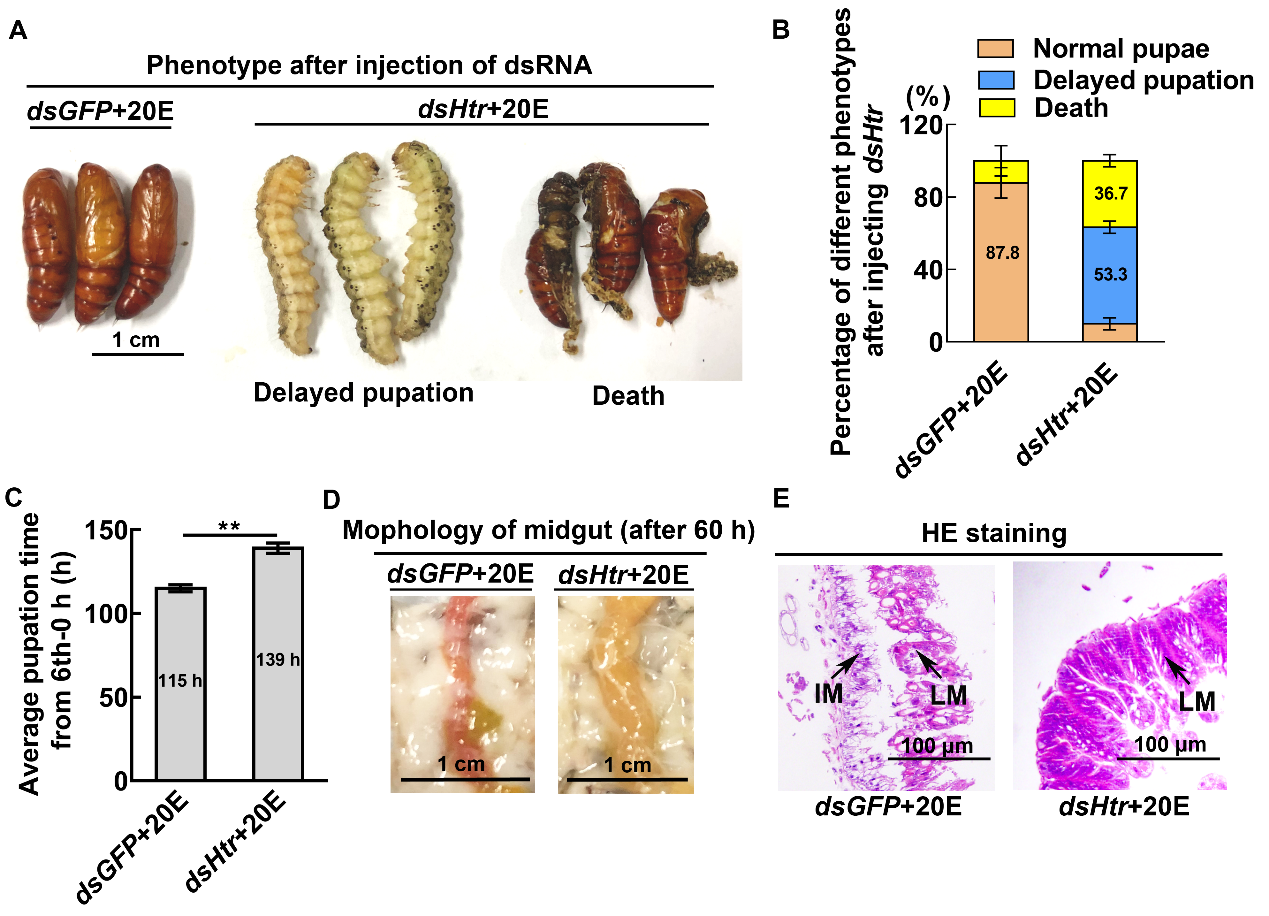


Supplementary Figure 5. Knockdown of *Htr* delayed larval-pupal transition and induced abnormal pupae. (**A)** Phenotypes after *dsHtr* or *dsGFP* injection (sixth instar 6 h larvae for the first dsRNA injection, thrice at 24 h intervals, 1 µg dsRNA/larva), and threated with 20E (500 ng/larva). Images were obtained at 120 h after the first dsRNA injection. Scale bar = 1 cm. **(B)** Percentages of the phenotypes in A. **(C)** Statistical analysis of pupation time from 6th instar 0 h larvae developing to pupae. **(D)** Morphology of the midgut 60 h after the first dsRNA injection. **(E)** HE-stained midgut after knockdown of *Htr*, observed at 60 h after the first dsRNA injection. The bars represent 100 μm. The experiments were performed in triplicate, and significant differences were calculated using Student’s *t*-test (***p* < 0.01). The bars indicate the mean ± SD.


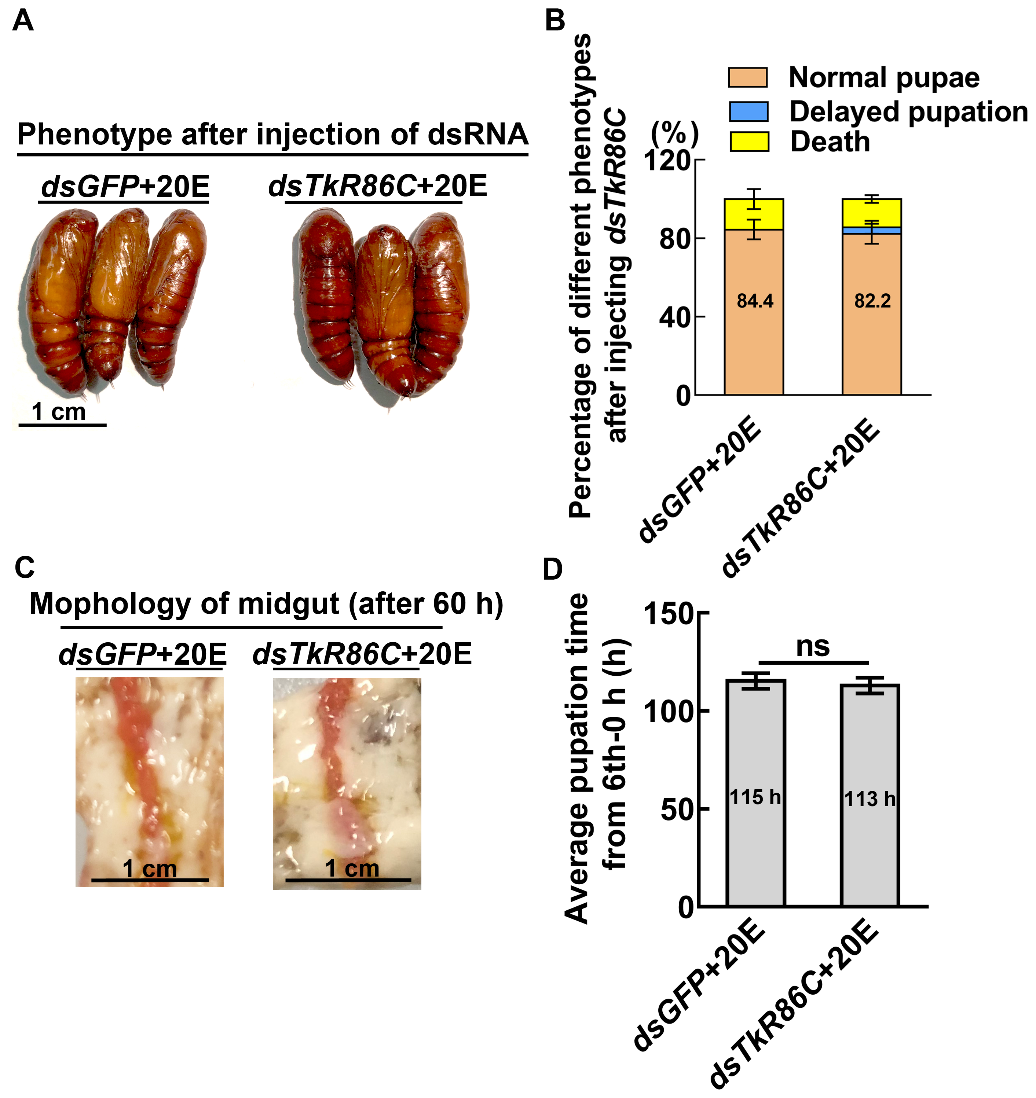


Supplementary Figure 6. Knockdown of *TkR86C* induced no changes in phenotype. (**A)** Phenotypes after *dsTkR86C* or *dsGFP* injection (sixth instar 6 h larvae for the first dsRNA injection, thrice at 24 h intervals, 1 µg dsRNA/larva), and treated with 20E (500 ng/larva). Images were obtained at 120 h after the first dsRNA injection. Scale bar = 1 cm. **(B)** Percentages of the phenotypes in A. **(C)** Morphology of the midgut 60 h after the first dsRNA injection. **(D)** Statistical analysis of pupation time from 6th instar 0 h larvae developing to pupae.


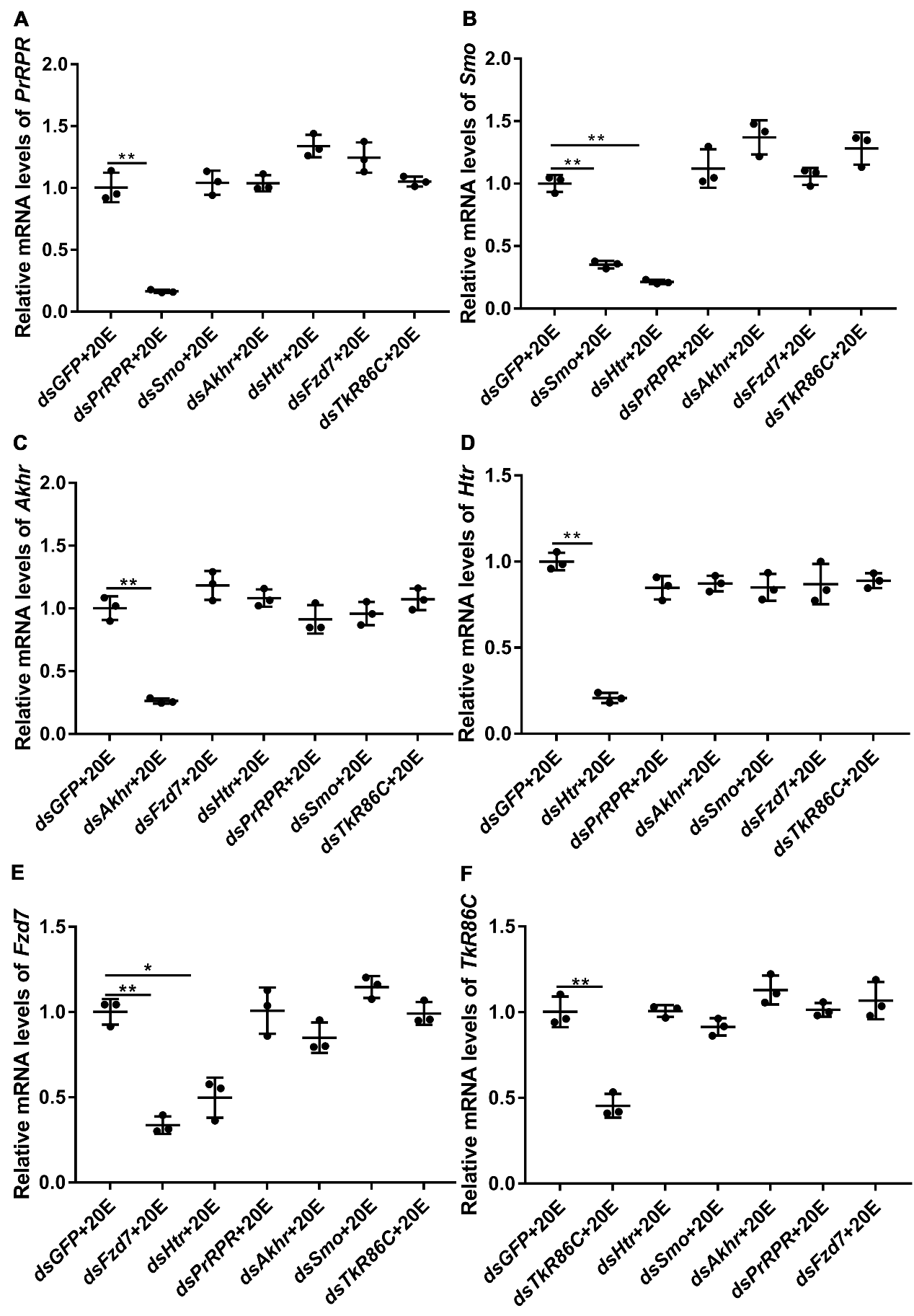


Supplementary Figure 7. qRT-PCR showing the off-target examination after knocking down GPCRs. (**A-E)** The relative mRNA levels of *PrRPR*, *Smo*, *Akhr*, *Htr*, *Fzd7*, and *TkR86C* after knockdown of these six GPCRs. The experiments were performed in triplicate and significant differences were calculated using Student’s *t*-test (**p* < 0.05; ***p* < 0.01). The bars indicate the mean ± SD.


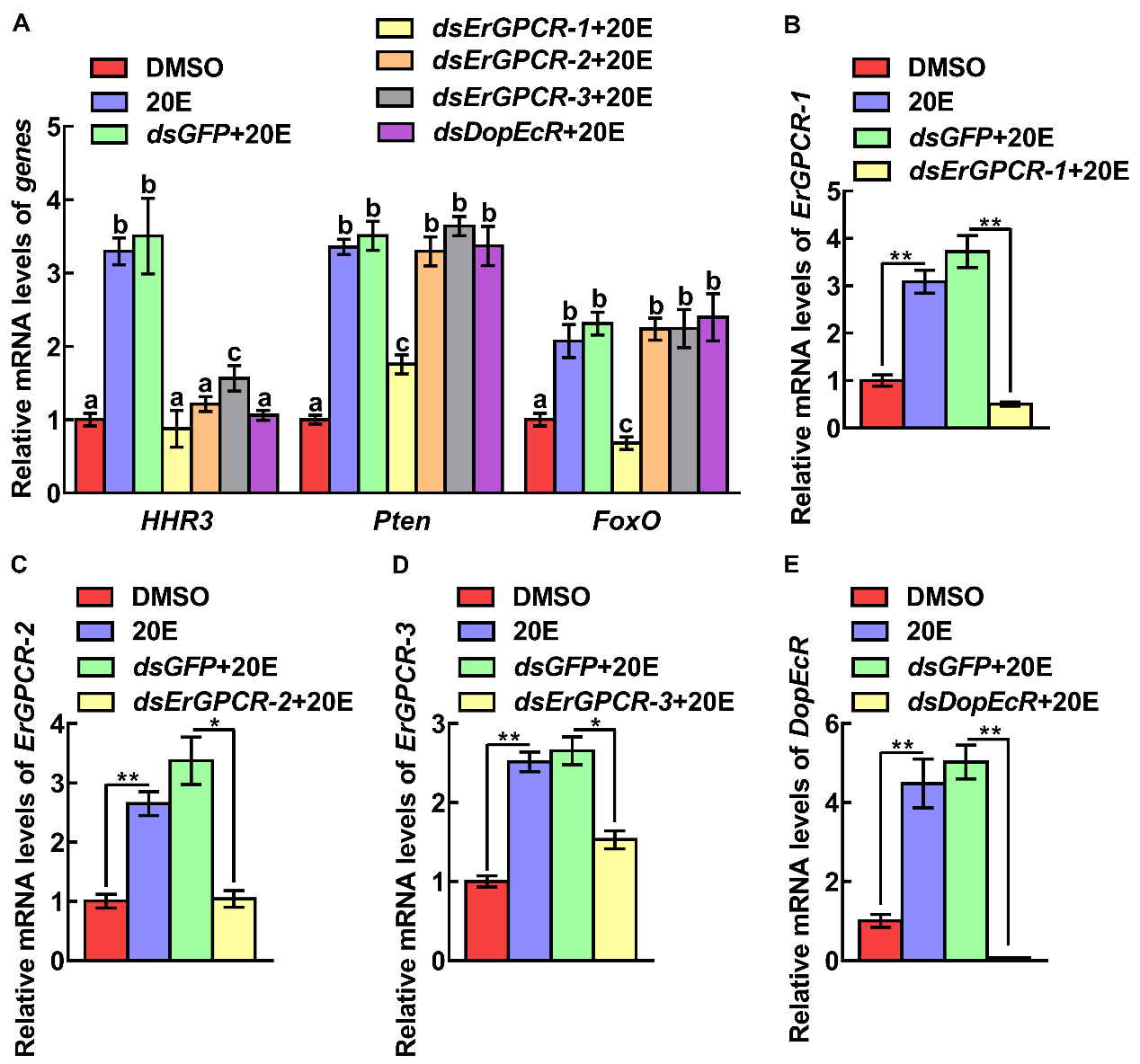


## **Supplementary Figure 8.** qRT-PCR showing the relative mRNA levels of genes after knocking down *ErGPCR-1*, *ErGPCR-2*, *ErGPCR-3*, and *DopEcR*. (A) The mRNA expression levels of *HHR3*, *Pten*, and *FoxO* in 6th-72 h larval midgut after *ErGPCR-1*, *ErGPCR-2*, *ErGPCR-3*, and *DopEcR* knockdown (sixth instar 6 h larvae for the first *dsRNA* injection, thrice at 24 h intervals, 1 µg *dsRNA*/larva, and treated with 20E (500 ng/larva) for 12 h). DMSO was used as the solvent control. *Actb* was used as the control gene. Statistical analysis was conducted using ANOVA, different letters represent significant differences (*p* < 0.05). The bars indicate the mean ± SD of three experiments. (B-E) The efficiency of *ErGPCR-1*, *ErGPCR-2*, *ErGPCR-3*, and *DopEcR* knockdown. The experiments were performed in triplicate and significant differences were calculated using Student’s *t*-test (**p* < 0.05; ***p* < 0.01). The bars indicate the mean ± SD.

## **Supplementary** Table 1. Classical GPCRs in the genome of *Helicoverpa armigera*

| **Classical GPCRs** | | | |
| --- | --- | --- | --- |
| **ErGPCR-1** | **LOC110383701** | **opsin-2** | **LOC110370149** |
| **ErGPCR-2** | **LOC110375440** | **Uvop-like** | **LOC110374920** |
| **Mth2-like2** | **LOC110382910** | **opsin-3** | **LOC110372004** |
| **Mth-like3-1** | **LOC110382950** | **Htr1a** | **LOC110375558** |
| **Mth2-like7** | **LOC110382857** | **moody-like2** | **LOC110384547** |
| **Mth-like1** | **LOC110383706** | **Tre1** | **LOC110375577** |
| **Mth2-like10** | **LOC110383675** | **moody** | **LOC110384506** |
| **Mth-like3-3** | **LOC110383699** | **moody-like3** | **LOC110384507** |
| **Mth2-like6** | **LOC110383700** | **NPFR** | **LOC110375929** |
| **Mth-like4** | **LOC110383702** | **PrRPR-like2** | **LOC110372006** |
| **Mth2-like9** | **LOC110383705** | **PrRPR** | **LOC110371999** |
| **Mth2-like5** | **LOC110383704** | **PrRPR-like1** | **LOC110372012** |
| **Mth2-like4** | **LOC110379106** | **RYa-R** | **LOC110384181** |
| **Mth-like3-2** | **LOC110379107** | **RYa-R-like1** | **LOC110383999** |
| **ErGPCR-3** | **LOC110372812** | **RYa-R-like2** | **LOC110375244** |
| **Fzd2** | **LOC110375987** | **OX1R-like1** | **LOC110376619** |
| **Fzd7** | **LOC110375464** | **OX1R-like2** | **LOC110376614** |
| **Fzd4** | **LOC110377715** | **TkR99D** | **LOC110384010** |
| **Fzd10** | **LOC110383311** | **TkR86C** | **LOC110378152** |
| **GPCR158** | **LOC110373055** | **TkR86C-2** | **LOC110380807** |
| **GPCR4** | **LOC110374861** | **Npffr1-like** | **LOC110376015** |
| **Mth-like** | **LOC110383703** | **Trhr** | **LOC110374305** |
| **Gabbr1** | **LOC110384642** | **TrissinR-like** | **LOC110380368** |
| **Gabbr2** | **LOC110372670** | **Sstr2** | **LOC110379454** |
| **Adgra3** | **LOC110372221** | **Rrh** | **LOC110373103** |
| **SIFaR** | **LOC110378403** | **Adora2b** | **LOC110372575** |
| **LOC110375868** | **LOC110375868** | **CCHa1-R** | **LOC110373200** |
| **GPR158-like** | **LOC110376110** | **CCHa2-R-like** | **LOC110379197** |
| **Fshr** | **LOC110383514** | **CCAP-R-like1** | **LOC110378296** |
| **PK1-R-like2** | **LOC110371637** | **CCAP-R-like2** | **LOC110380985** |
| **PK1-R-like1** | **LOC110371629** | **Oct-beta1R-like** | **LOC110374292** |
| **CapaR** | **LOC110372982** | **Oct-beta3R-like** | **LOC110374308** |
| **CapaR-like1** | **LOC110380327** | **Oct-beta2R-like** | **LOC110374293** |
| **Cckbr-like** | **LOC110370995** | **Dop2-like** | **LOC110382458** |
| **parapin opsin-like** | **LOC110372148** | **DopD2R-like** | **LOC110379565** |
| **Calcrl-like** | **LOC110374603** | **Oamb** | **LOC110379177** |
| **Calcrl** | **LOC110380875** | **Htr1** | **LOC110374913** |
| **CapaR-like2** | **LOC110380388** | **Htr** | **LOC110375681** |
| **Sstr4-like** | **LOC110379380** | **Htr1-like** | **LOC110380291** |
| **FMRFaR-like** | **LOC110382738** | **OctR** | **LOC110378428** |
| **Nmur2-like1** | **LOC110371660** | **TyrR** | **LOC110370301** |
| **opsin** | **LOC110375804** | **Grm2-like1** | **LOC110380183** |
| **Nmur2-like 2** | **LOC110384405** | **Grm2-like2** | **LOC110383234** |
| **Ptger1** | **LOC110378655** | **Smo** | **LOC110375416** |
| **Taar1** | **LOC110377506** | **DopEcR** | **LOC110374281** |
| **SPR** | **LOC110371493** | **OctR-like** | **LOC110379646** |
| **LOC110373470** | **LOC110373470** | **OctR1-like** | **LOC110379294** |
| **Mth-like5** | **LOC110379110** | **Htr2b** | **LOC110371148** |
| **Cirl** | **LOC110372419** | **No18** | **LOC110384550** |
| **DhR** | **LOC110370558** | **mAChR-DM1** | **LOC110376058** |
| **Tyra-2** | **LOC110380463** | **mAChR-M2** | **LOC110380854** |
| **Htr1a-like** | **LOC110373498** | **moody-like1** | **LOC110384572** |
| **Gnrhr** | **LOC110374373** | **Galr3** | **LOC110377545** |
| **Akhr** | **LOC110384063** | **stan** | **LOC110375009** |
| **Gnrhr-like** | **LOC110384307** | **Dop1-like** | **LOC110382322** |
| **SPR-like1** | **LOC110376398** | **Hrh2-like** | **LOC110373025** |
| **SPR-like4** | **LOC110376411** |  |  |
| **SPR-like5** | **LOC110376412** | **GPCRs uninvolved in trees** | |
| **SPR-like2** | **LOC110370003** | **RYa-R-like3** | **LOC110375089** |
| **SPR-like3** | **LOC110369979** | **Mth2-like3** | **LOC110383697** |
| **opsin-1** | **LOC110378494** | **LOC110372829** | **LOC110372829** |
| **opsin-1-like** | **LOC110370495** | **Mth2-like8** | **LOC110374712** |

## **Supplementary** Table 2. Primers used in this study.

| Primer names | Primer sequences 5’-3’ |
| --- | --- |
| **qRT-PCR** |  |
| *GPCR4*-F | tgactaacgctgaacccg |
| *GPCR4*-R | tcgttgttgtccgattgg |
| *Fshr*-F | gtgaacaatacgctcggttc |
| *Fshr*-R | cgtcttcgcacggattcagc |
| *Fzd7*-F | tactgtttaccctggtgatacttt |
| *Fzd7*-R | cgtcttcttgtttagcgtgat |
| *Htr*-F | gaggaaagcggcgaaaaca |
| *Htr*-R | gtagcccagccacagcgag |
| *PrRPR*-F | gtcgctcttgacatccacag |
| *PrRPR*-R | ctccatcgtcttcatacacc |
| *Rya-R*-F | cgccaaccgcatcactat |
| *Rya-R*-R | cacgccttcaggaaactc |
| *NPFR*-F | tccgctcctatacgggtggtt |
| *NPFR*-R | tcttcctgctctttatcctc |
| *Smo*-F | gcttccgtgctttgggtaa |
| *Smo*-R | gccatcgtcgctatcgtga |
| *Akhr*-F | gacgaactgcctctggacat |
| *Akhr*-R | agcaccgtcaagttccctgt |
| Galr3-F | tcaacgctccgctccagacg |
| Galr3-R | cctaatgcacccgccaccac |
| *TkR86C*-F | gagaaaggtggtgaggatgt |
| *TkR86C*-R | gccaatagaagaagaggtag |
| *Fzd4*-F | cagtttggtgggtggtggta |
| *Fzd4*-R | agtgaggagtggcggttgtt |
| *Opsin*-F | atcgcaaatccccttcccca |
| *Opsin*-R | tccctgcaacgccaatcact |
| *HHR3*-F | tcaagcacctcaacagcagcccta |
| *HHR3*-R | gactttgctgatgtcaccctccgc |
| *Pten*-F | tcttccacttctggttca |
| *Pten*-R | gtgtttatgctgcttatcc |
| *β-actin*-F | cctggtattgctgaccgtatgc |
| *β-actin*-R | ctgttggaaggtggagagggaa |
| *FoxO*-F | tcattacccaagccagcac |
| *FoxO*-R | tccatccagccgaagagt |
| **RNAi** |  |
| *GFP*-RNAi-F | gcgtaatacgactcactataggtggtcccaattctcgtggaac |
| *GFP*-RNAi-R | gcgtaatacgactcactataggcttgaagttgaccttgatgcc |
| *Fzd7*-RNAi-F | gcgtaatacgactcactataggatacagttcaaggtgccg |
| *Fzd7*-RNAi-R | gcgtaatacgactcactataggatgacccaccaaatgctc |
| *Htr*-RNAi-F | gcgtaatacgactcactatagggcacctctgctgggatgg |
| *Htr*-RNAi-R | gcgtaatacgactcactataggaatgttttcgccgctttc |
| *PrRPR*-RNAi-F | gcgtaatacgactcactataggacattcagcacaaccagc |
| *PrRPR*-RNAi-R | gcgtaatacgactcactataggcgaacgccttacggattt |
| *Smo*-RNAi-F | gcgtaatacgactcactataggggaatctgtttcgtgggt |
| *Smo*-RNAi-R | gcgtaatacgactcactataggcatcagggtagtcagtaggc |
| *Akhr*-RNAi-F | gcgtaatacgactcactataggtcataccgttgctgtccact |
| *Akhr*-RNAi-R | gcgtaatacgactcactatagggaagtcttgaaccacgcctc |
| *TkR86C*-RNAi-F | gcgtaatacgactcactataggagtcgtgggaccatctca |
| *TkR86C*-RNAi-R | gcgtaatacgactcactataggtatgtcacagccaggaac |
| **Overexpression** |  |
| PRRPR-oex-F | tactcagagctcatgaccgacctatgggtt |
| PRRPR-oex-R | tactcagtcgaccagcgcagacacgatagg |
| SMO-oex-F | tactcacaattggatgaccccttggcgatgg |
| SMO-oex-R | tactcagtcgacgtcgaagctgaacttgac |
